# Minimizing Paravalvular Regurgitation With the Novel SAPIEN 3 Ultra TAVR Prosthesis: A Real-World Comparison Study

**DOI:** 10.3389/fcvm.2021.623146

**Published:** 2021-03-18

**Authors:** Alexander R. Tamm, Michaela M. Hell, Martin Geyer, Felix Kreidel, Jaqueline G. da Rocha e Silva, Meike Seidl, Tobias F. Ruf, Angela Kornberger, Andres Beiras-Fernandez, Thomas Münzel, Ralph Stephan von Bardeleben

**Affiliations:** ^1^Center of Cardiology, University Medical Center Mainz, Mainz, Germany; ^2^Department of Cardiac and Vascular Surgery, University Medical Center Mainz, Mainz, Germany

**Keywords:** transcatheter aortic valve replacement, balloon expandable valve, paravalvular regurgitation, SAPIEN 3 ultra, aortic stenosis

## Abstract

**Objectives:** We investigated performance and outcome of the latest-generation balloon-expandable SAPIEN 3 Ultra prosthesis (S3U) compared to the established SAPIEN 3 prosthesis (S3) in a real-world cohort, with focus on paravalvular regurgitation (PVR).

**Background:** PVR is an adverse prognostic indicator of short- and long-term survival after transcatheter aortic valve replacement (TAVR). The S3U has been designed to improve sealing.

**Methods:** We enrolled 343 consecutive patients presenting with severe native aortic valve stenosis eligible for a balloon-expandable prosthesis. The established S3 was implanted in the first 200 patients, the following 143 patients received the novel S3U after introduction in our institution. Primary endpoint was PVR after TAVR. Furthermore, we investigated procedural parameters and in-hospital and 30-day outcome.

**Results:** PVR was significantly lower in the S3U cohort compared to the S3 cohort. They differed in their rate of mild PVR (11.2 vs. 48.0%, *p* < 0.001), whereas at least moderate PVR was similarly low in both cohorts (0.7 vs. 0.5%, *p* = 0.811). A significant reduction of post-dilatation rate, fluoroscopy time, and amount of contrast was observed in patients treated with the novel S3U (*p* < 0.001). The rate of adverse events in the in-hospital course and at 30 days were similarly low. At 30 days more patients receiving S3U improved in NYHA class (improvement ≥2 grades 34.6 vs. 19.9%, *p* = 0.003).

**Conclusion:** The current study provides evidence that the novel S3U strongly minimizes PVR, thereby demonstrating the efficacy of improved sealing. Further studies will have to address if the observed reduction of PVR with S3U has prognostic significance.

## Introduction

Transcatheter aortic valve replacement (TAVR) has evolved into a standard treatment for patients with symptomatic severe aortic valve stenosis (AS) regardless of surgical risk (1, 2). With respect to the hemodynamic status, TAVR offers lower transvalvular aortic gradients, larger effective orifice area, and a lower rate of patient-prosthesis mismatch compared to a surgical prosthesis (3, 4). However, paravalvular regurgitation (PVR) occurs more often in TAVR procedure and has been associated with a worse short- and long-term survival (4–7). With the advancement in prosthetic valve technology, the incidence of at least moderate PVR has significantly declined. Nevertheless, several reports suggested that even mild PVR is an adverse prognostic indicator for mid- and long-term outcome (6–9).

Different balloon- and self-expandable prosthesis systems have been released and approved for clinical use. Due to their mechanical characteristics, balloon-expandable prostheses are less prone to PVR compared to self-expandable prostheses (10). Among the balloon-expandable devices, the SAPIEN series (Edwards Lifesciences Inc., Irvine, CA, USA) has been recently extended with the novel S3U. It features ~40% taller, textured polyethylene terephthalate outer skirt compared to the third-generation S3, intended to improve sealing of the prosthesis and reduce the amount of PVR. First reports with the S3U system showed a good in-hospital and 30-day clinical outcome but the quality of comparison is limited (11–13).

The aim of this real-world, high-volume TAVR single-center observer study was to compare the procedural performance and 30-day outcome of the latest-generation balloon-expandable prosthesis—the S3U—with its preceding model, the S3. The particular focus and primary endpoint of the study was the rate of PVR.

## Methods

### Patient Population and Pre-interventional Assessment

We screened all consecutive patients with high-grade symptomatic native AS scheduled for transfemoral TAVR and eligible for a balloon-expandable prosthesis between January 2019 and March 2020 for enrolment. Non-eligibility for a balloon-expandable but rather self-expanding prosthesis was the presence of moderate to severe annular or left ventricular outflow tract calcifications due to a higher risk for annulus rupture with balloon-expandable devices. Furthermore, patients with severely calcified and narrow femoral arteries were in favor for a self-expandable prosthesis (Evolut R, Medtronic Inc., Minneapolis, MN, USA) due to the smaller absolute required vessel diameter of the corresponding access sheath. Patients with a large annulus size suitable for the 29 mm SAPIEN prosthesis were not enrolled in the study, as the S3U is only available in sizes 20, 23, and 26 mm. Furthermore, since no 20 mm S3U has been implanted, the two patients receiving a 20 mm S3 were excluded in the comparison.

**Figure 1:**
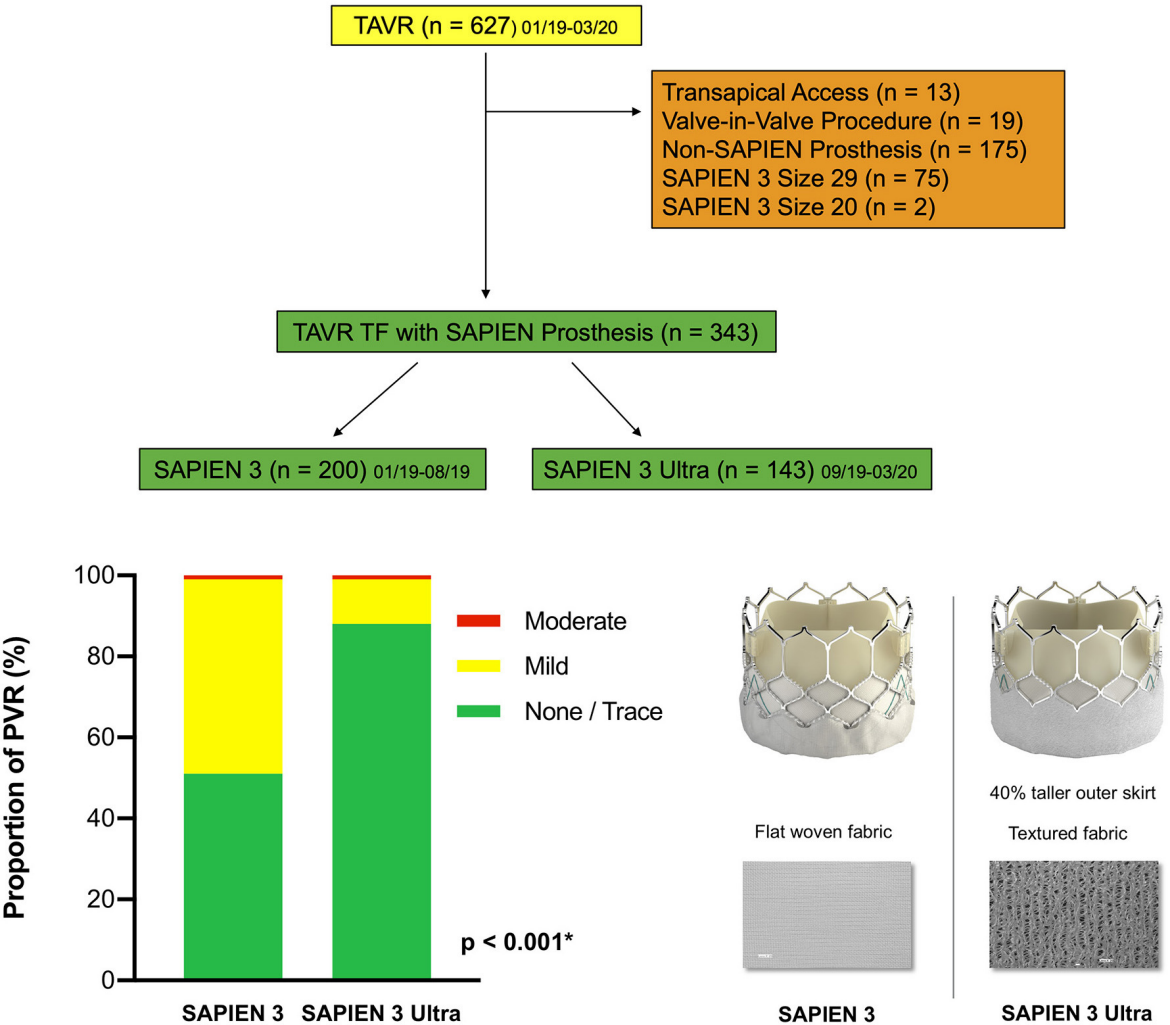
Patient population. A total of 627 patients undergoing TAVR from January 2019 until March 2020 at the Heart Valve Center Mainz were screened. Reasons for exclusion are displayed on the right side. Finally, 200 patients with a SAPIEN 3 and 143 patients with a SAPIEN 3 Ultra were enrolled in the study. TAVR, transcatheter aortic valve replacement; TF, transfemoral access. Central illustration. Impact on paravalvular regurgitation: The SAPIEN 3 Ultra significantly lowers the rate of paravalvular regurgitation after TAVR compared to the SAPIEN 3. The SAPIEN 3 (left) and last-generation SAPIEN 3 Ultra prostheses (right). The novel SAPIEN 3 Ultra features a taller, textured polyethylene terephthalate outer skirt with ~40% increase in height. All other prosthesis components remained unchanged from the SAPIEN 3. PVR, paravalvular regurgitation. *P*-value was calculated by Chi squared test. Data represented as percentage of total.

The S3 has been implanted at our institution in more than 1,000 patients since 2014. In September 2019, we switched to the latest-generation S3U. Therefore, in the first half of the enrolment period (January–August 2019), all patients received a S3 (200 patients). Starting from September 2019, all further patients were implanted the latest-generation S3U. Finally, 343 patients were included in the study (Figure 1). The study was approved by the Institutional Review Board (reference number: 2019-14692), all patients provided written consent. Covariates, including cardiac history and risk factors, were obtained by a structured interview. The “European System for Cardiac Operative Risk Evaluation II” (EuroSCORE II) and the “Society of Thoracic Surgeon” (STS) score were calculated in all patients. Indication for TAVR was confirmed by the interdisciplinary heart team.

In all patients, severe AS (aortic valve orifice area <1.0 cm^2^) was diagnosed in our echocardiography lab. Pre-interventional CT imaging of the aortic annulus and the vascular access route was performed according to current recommendations and post-processed using a semi-automated software (3mensio Structural Heart, Pie Medical Imaging BV, Maastricht, The Netherlands) (14, 15). Aortic root dimensions, distance between annular plane and coronary artery ostia, an orthogonal fluoroscopic angulation plane for implantation and the vascular access route were assessed in all patients. Additionally, the distribution of annular/sub-annular and left ventricular outflow tract calcification was qualitatively graded (none, mild, moderate, and severe) (14).

### S3U and Delivery System

The CE-mark approved S3U is available in three sizes: 20, 23, and 26 mm. The novel key feature of the S3U is a taller, textured polyethylene terephthalate outer skirt with ~40% increase in height compared to the third-generation S3 which is supposed to improve the sealing of the prosthesis (Figure 1). Other components of the valve, including the cobalt-chromium alloy stent with a low delivery profile, open cell geometry and high radial strength, the bovine pericardial leaflets and the inner sealing skirt remain unchanged to the S3. According to manufacturer's recommendations, the Commander delivery system with the 14 French eSheath was applied.

### Implantation Procedure

The TAVR procedure was performed under sedation or, if needed, in intubation anesthesia in a hybrid operating suite. The interdisciplinary established team of interventional cardiologists and cardiac surgeons has been trained and certified for the SAPIEN system and performed more than 1,000 previous joint TAVR procedures according to current recommendations. Apart from the prosthesis type, the technical aspects of the procedure did not differ between both cohorts and followed the recommended standards. Prosthesis sizing was based on pre-procedural CT analysis according to manufacturers' recommendations. A transvenous temporary pacemaker was applied in all patients. Pre-dilatation was at discretion of the interventionalist and mainly due to severe commissural calcification or very high transvalvular gradients. Post-dilatation was performed in patients with at least moderate residual PVR evaluated by multi-modality assessment (angiography, transthoracic echocardiography, and invasive hemodynamic assessment with simultaneous determination of left ventricular and aortic pressures). The puncture site was sealed using dedicated closure systems. Procedural parameters including procedure time (duration from puncture to suture), need for post-dilatation, amount of contrast and fluoroscopy time were assessed. Prosthesis oversizing was calculated with the formula (S3U or S3 nominal area/MDCT systolic annular area−1) × 100 (16) and four categories were set stepwise from <0 to >10% (Supplementary Table 2) according to the literature (17, 18).

### Post-procedural Course

Post-procedurally, the temporary pacemaker sheath remained for 24 h and patients were ECG-monitored through 72 h. Indications for a permanent pacemaker implantation (PPI) were a third-degree atrioventricular block or a symptomatic second-degree atrioventricular block Mobitz II.

A 3-month dual antiplatelet therapy followed by a lifelong single antiplatelet therapy was prescribed as antithrombotic treatment. Patients with the indication for oral anticoagulation received a 1-month single antiplatelet therapy in addition to their lifelong oral anticoagulation.

Pre-discharge transthoracic echocardiography was performed in all patients for semi-quantitative grading of final PVR, determination of the prosthesis gradient and cardiac function. According to current recommendations, PVR was graded as none or trace, mild, moderate and severe (19).

### Outcome

Study endpoints were established according to the Valve Academic Research Consortium-2 (VARC-2) definitions (20). For in-hospital outcome, we included device success, all-cause death, cardiac death, myocardial infarction, stroke, vascular complications, bleeding, new PPI, PVR grade at discharge and acute kidney injury in our analysis. Patients were followed up in an outpatient department at 30 days or by telephone contact for assessment of the early safety combined endpoint.

### Statistics

Statistical analysis was performed using SPSS software (IBM® SPSS® statistics, version 24 for Mac). Continuous variables were expressed as mean ± SD when normally distributed, otherwise as median and interquartile ranges. Categorical variables were presented as frequencies and percentage, unless otherwise specified. Shapiro–Wilk test was used to assess normality for continuous data. Statistical significance was assessed using a *t*-test in normally distributed data or a Mann-Whitney-*U*-test in non-normally distributed data. Chi-square test was used to compare categorical variables. For multi-variate analysis, stepwise logistic regression models were used to examine independent relationships between patient and procedural parameters and PVR or PPI. All statistical tests were two-sided and *p* < 0.05 was considered to be statistically significant.

## Results

### Baseline Characteristics

The total cohort included 343 patients (43.1% male) with a mean patient age of 80 ± 7 years and a mean EuroSCORE II of 5.2 ± 5.2% and STS Score 5.0 ± 4.1%, respectively. Except for a higher rate of prior myocardial infarction and less patients with NYHA class III in the S3 cohort, there were no significant differences in terms of age, cardiovascular risk factors or comorbidities between patients enclosed in the S3 (200 patients) and the S3U (143 patients) cohorts. Baseline characteristics are presented in Table 1.

**Table 1:** Patient characteristics.

	**All patients (*N* = 343)**	**S3 (*N* = 200)**	**S3U (*N* = 143)**	***p*-value**
Age (years)	80.2 ± 7.1	79.9 ± 7.3	80.7 ± 6.8	0.297
Male sex—*n* (%)	148 (43.1)	85 (42.5)	63 (44.1)	0.774
BMI(kg/m^2^)	27.1 ± 4.9	27 ± 5.3	27.3 ± 4.3	0.561
EuroSCORE II	5.2 ± 5.2	5.4 ± 5.6	5 ± 4.4	0.493
STS score	5.0 ± 4.1	5.0 ± 4.1	5.1 ± 4.2	0.771
*NYHA class*				
I	15 (4.4)	10 (5.0)	5 (3.5)	0.502
II	77 (22.4)	51 (25.5)	26 (18.2)	0.109
III	229 (66.8)	123 (61.5)	106 (74.1)	0.014
IV	22 (6.4)	16 (8.0)	6 (4.2)	0.156
Coronary artery disease—*n* (%)	254 (60.3)	176 (63.3)	78 (54.5)	0.082
Previous myocardial infarction—*n* (%)	47 (13.7)	34 (17.0)	13 (9.1)	0.036
Previous PCI—*n* (%)	157 (45.8)	94 (47.0)	63 (44.1)	0.589
Previous open-heart surgery—*n* (%)	30 (8.7)	16 (8.0)	14 (9.8)	0.563
Previous stroke—*n* (%)	42 (12.2)	23 (11.5)	19 (13.3)	0.619
Peripheral artery disease ≥ grade II	33 (9.6)	21 (10.5)	12 (8.4)	0.514
Arterial hypertension—*n* (%)	309 (90.1)	176 (88.0)	133 (93.0)	0.126
Diabetes (%)	100 (29.2)	58 (29.0)	42 (29.4)	0.941
COPD ≥ grade II—*n* (%)	21 (6.1)	11 (5.5)	10 (7.0)	0.57
GFR (ml/min)	55.6 ± 23.8	55.7 ± 23.1	55.5 ± 24.8	0.932
Reduced GFR <30 ml/min	41 (12.0)	22 (11.0)	19 (13.3)	0.52
Congenital bicuspid valve	49 (14.3)	29 (14.5)	20 (14.0)	0.893
Atrial fibrillation—*n* (%)	84 (24.6)	47 (23.6)	37 (26.1)	0.606
Permanent pacemaker—*n* (%)	38 (11.1)	19 (9.5)	19 (13.3)	0.271
Preexisting RBBB	21 (6.1)	14 (7.0)	7 (4.9)	0.423
Pulmonary hypertension—*n* (%)	50 (14.6)	34 (17.0)	16 (11.2)	0.133
Left ventricular ejection fraction (%)	55.2 ± 10.3	55.5 ± 10.3	54.8 ± 10.2	0.522
Reduced Ejection Fraction <40%	34 (10.0)	19 (9.5)	15 (10.6)	0.758
Aortic valve area (cm^2^)	0.76 ± 0.18	0.75 ± 0.18	0.77 ± 0.18	0.322
Aortic valve peak gradient (mmhg)	62.1 ± 26.5	63.8 ± 28.9	59.7 ± 22.6	0.155
Aortic valve mean gradient (mmhg)	37.1 ± 14.3	37.4 ± 14.1	36.8 ± 14.5	0.701
Severe commissural calcification—*n* (%)	139 (40.6)	79 (39.7)	60 (42.0)	0.675

*BMI, Body Mass Index; EuroSCORE, European System for Cardiac Operative Risk Evaluation; STS Score, Society of Thoracic Surgeons Predicted Risk of Mortality; NYHA class, New York Heart Association Functional Classification of Heart Failure; PCI, Percutaneous Coronary Intervention; CABG, Coronary Artery Bypass Graft; TIA, Transient Ischemic Attack; COPD, Chronic Obstructive Pulmonary Disease; GFR, Glomerular filtration rate; ICD, Internal Cardioverter Defibrillator; RBBB, Right Bundle Branch Block, S3, SAPIEN 3, S3U, SAPIEN 3 Ultra*.

*P-value was calculated by Chi squared test and Student's t-test. Data represented as mean ± SD for metric variables and number and percentage of total in categorical variables*.

### Procedural Data

Device success by VARC2-criteria was achieved in 95.6% patients with no significant difference between the two cohorts (*p* = 0.35). There was no peri-procedural death or incorrect positioning of the prosthesis with the need for implantation of a second valve. The 15 cases not meeting the VARC2-criteria of device success had either a moderate PVR after initial valve deployment (2 patients, 0.5%) or a mean gradient ≥20 mmHg as assessed by echocardiography at discharge (13 patients, 3.8%).

**Figure 2:**
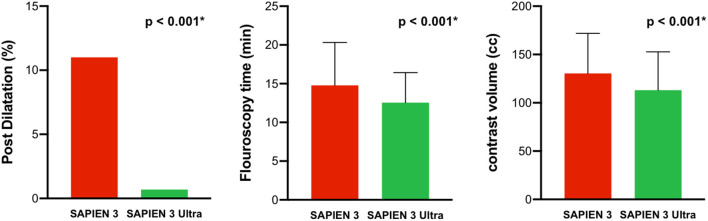
Procedural performance. The SAPIEN 3 Ultra performed significantly better in terms of post dilatation rate, fluoroscopy time, and amount of contrast compared to the SAPIEN 3. *P*-value was calculated by Chi squared test and Student's *t*-test. Data represented as mean ± SD for metric variables and number and percentage of total in categorical variables.

Among the patients receiving the latest-generation S3U, a 23 mm prosthesis was implanted in 70 patients (49%) and a 26 mm prosthesis in 73 patients (51%). In the third-generation S3 cohort, 106 patients (53%) received a 23 mm prosthesis and 94 patients (47%) a 26 mm prosthesis. Pre-dilatation was performed in 36 patients (10.5%) with no significant difference between both cohorts (*p* = 0.998). Likewise, procedural time was similar between both treatment groups (*p* = 0.43). However, post-dilatation due to an at least moderate post-implant PVR after valve deployment was significantly lower in the S3U cohort compared to the S3 cohort (1 vs. 22 patients, *p* < 0.001). Furthermore, fluoroscopy time and amount of contrast were significantly lower in the S3U cohort compared to the S3 cohort (12.6 ± 3.9 vs. 14.8 ± 5.5 min, *p* < 0.001, and 113.1 ± 39.6 vs. 130.4 ± 41.6 ml, *p* < 0.001, respectively, Figure 2). Major TAVR-related complications occurred in 3 patients (all cardiac tamponades due to wire related ventricular injury, one of them with need for median sternotomy in the S3U cohort, 2 with pericardiocentesis alone in the S3 cohort). There was no peri-procedural death. Procedural data are summarized in Table 2.

**Table 2:** Procedural details.

	**All patients (*N* = 343)**	**S3 (*N* = 200)**	**S3U (*N* = 143)**	***p*-value**
Prosthesis size—*n* (%)				
23 mm	176 (51.3)	106 (53.0)	70 (49.0)	0.459
26 mm	167 (48.7)	94 (47.0)	73 (51.0)	
Balloon pre-dilatation—*n* (%)	36 (10.5)	21 (10.5)	15 (10.5)	0.998
Balloon post-dilatation—*n* (%)	23 (6.7)	22 (11.0)	1 (0.7)	<0.001
General anesthesia—*n* (%)	45 (13.1)	32 (16.0)	13 (9.1)	0.062
Procedure time (min)	64.6 ± 33.9	65.8 ± 20.2	62.9 ± 46.9	0.431
Fluoroscopy time (min)	13.9 ± 5.0	14.8 ± 5.5	12.6 ± 3.9	<0.001
Contrast volume (cc)	123.3 ± 41.6	130.4 ± 41.6	113.1 ± 39.6	<0.001

*S3, SAPIEN 3; S3U, SAPIEN 3 Ultra*.

*P-value was calculated by Chi squared test and Student's t-test. Data represented as mean ± SD for metric variables and number and percentage of total in categorical variables*.

### Outcome

The primary endpoint PVR was significantly lower in the S3U cohort compared to the S3 cohort (11.9 vs. 48.5%, *p* < 0.001, Figure 1). There was no patient with a severe PVR in both cohorts. The rate of moderate PVR was very low (0.6%) and was observed in 1 patient in the S3U and 1 in the S3 cohort, respectively. In contrast, the rate of mild PVR was significantly lower in patients receiving a S3U compared to patients receiving the third-generation prosthesis (11.2 vs. 48%, *p* < 0.001). In age- and sex-adjusted multi-variable analysis (Supplementary Table 3), prosthesis type and oversizing (odds ratio, OR 0.75, *p* = 0.002) remained the only two independent predictors for the presence of PVR with the S3U having a more than 7-fold reduced risk (OR 0.13, *p* < 0.001).

In-hospital outcome showed no significant difference in all cause death, stroke, acute myocardial infarction, vascular complication, bleeding complication, acute kidney injury, need for PPI, new onset of atrial fibrillation or mean prosthetic gradient as assessed by echocardiography.

**Figure 3:**
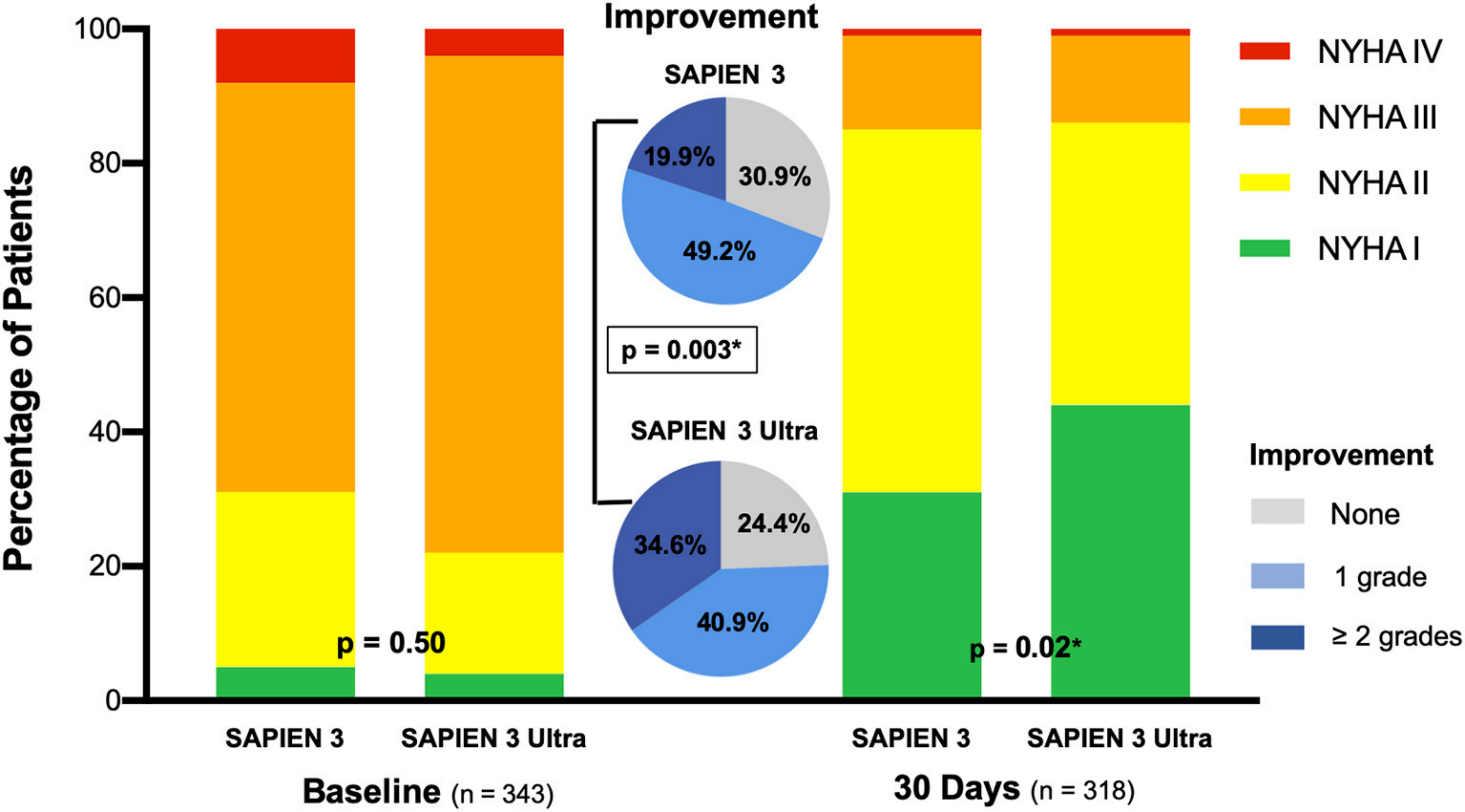
NYHA functional class. NYHA (New York Heart Association) functional class at baseline and 30 days. Significantly more patients receiving the SAPIEN 3 Ultra were free of symptoms and improved in NYHA class at 30 days compared to patients with the SAPIEN 3. *P*-value was calculated by Chi squared test. Data represented as percentage of total.

For 30-day outcome assessment, 17 (4.9%) patients were lost to follow-up. Early safety end-point including, among other parameters, all cause death (1.8%) and stroke (1.9%), revealed no significant difference between the S3U and S3 cohort (3.9 vs. 4.6%, *p* = 0.76). Patients who were implanted a S3U were significantly more often free of symptoms (NYHA class I, 44.1 vs. 31.4%, *p* = 0.02) and improvement of two or more NYHA class grades was higher (34.6 vs. 19.9%, *p* = 0.003) compared to patients with a S3, while overall improvement (reduction of at least 1 NYHA class) did not show a significant difference (75.6 vs. 69.1%, *p* = 0.21) (Figure 3).

The rate of new PPI was low in our study (8.9% at in-hospital and 9.6% at 30-day assessment) with no significant difference at in-hospital or 30-day post-procedural course between the two cohorts (*p* = 0.10 and *p* = 0.23, respectively). Age- and sex-adjusted multivariate analysis (Supplementary Table 4) including prosthesis type, severe commissural calcification, bicuspid anatomy, post-dilatation, right bundle branch block (RBBB), 1st degree AV Block, left bundle branch block (LBBB) and prosthesis oversizing revealed only a pre-procedural RBBB as a significant independent predictor for new PPI after TAVR (OR 9.22, *p* < 0.001). Peri- and post-procedural outcomes are summarized in Table 3.

**Table 3:** Peri- and post-procedural outcomes.

	**All patients (*N* = 343)**	**S3 (*N* = 200)**	**S3U (*N* = 143)**	***p*-value**
**In hospital**				
Device success—*n* (%)	328 (95.6)	193 (96.5)	135 (94.4)	0.350
All cause death—*n* (%)	2 (0.6)	1 (0.5)	1 (0.5)	0.846
Stroke—*n* (%)	4 (1.2)	2 (1.0)	2 (1.4)	0.735
Acute myocardial infarction—*n* (%)	1 (0.3)	1 (0.5)	0 (0.0)	0.397
*Vascular complications—*n* (%)*				
Minor	34 (9.9)	21 (10.5)	13 (9.1)	0.667
Major	5 (1.5)	2 (1.0)	3 (2.1)	0.403
*Bleeding complications—*n* (%)*				
Minor	12 (3.5)	5 (2.5)	7 (4.9)	0.234
Major	1 (0.3)	0 (0.0)	1 (0.7)	0.417
Life-threatening or disabling	1 (0.3)	0 (0.0)	1 (0.7)	0.469
Acute kidney injury ≥2—*n* (%)	7 (2.0)	5 (2.5)	2 (1.4)	0.477
New permanent pacemaker—*n* (%)	27/305 (8.9)	20/181 (11.0)	7/124 (5.6)	0.103
New atrial fibrillation—*n* (%)	4/239 (1.7)	2/140 (1.4)	2 / 99 (2.0)	0.826
*Paravalvular regurgitation—*n* (%)*				
None or trace	229 (66.8)	103 (51.5)	126 (88.1)	<0.001
Mild	112 (32.7)	96 (48.0)	16 (11.2)	<0.001
Moderate	2 (0.6)	1 (0.6)	1 (0.6)	0.811
Severe	0 (0.0)	0 (0.0)	0 (0.0)	1.000
Prosthetic mean gradient (mmHg)	11.6 ± 4.4	11.6 ± 4.7	11.7 ± 3.9	0.793
23 mm valve	12.4 ± 4.5	12.5 ± 4.8	12.2 ± 4.0	0.627
26 mm valve	10.6 ± 3.7	10.2 ± 3.7	11.1 ± 3.7	0.135
**At 30 days**				
All cause death—*n* (%)	6/326 (1.8)	3/197 (1.5)	3/129 (2.3)	0.598
Early safety—*n* (%)	14/326 (4.3)	9/197 (4.6)	5/129 (3.9)	0.763
Stroke—*n* (%)	6/326 (1.8)	4/197 (2.0)	2/129 (1.6)	0.752
New permanent pacemaker—*n* (%)	28/292 (9.6)	20/178 (11.2)	8/114 (7.0)	0.232
NYHA class—*n* (%)				
I	116/318 (36.5)	60/191 (31.4)	56/127 (44.1)	0.021
II	152/318 (49.1)	100/191 (53.4)	52/127 (42.5)	0.057
III	42/318 (13.5)	27/191 (14.1)	15/127 (12.6)	0.695
IV	3/318 (0.9)	2/191 (1.0)	1/127 (0.8)	0.814

*NYHA class, New York Heart Association Functional Classification of Heart Failure; S3, SAPIEN 3; S3U, SAPIEN 3 Ultra*.

*P-value was calculated by Chi squared test and Student's t-test. Data represented as mean ± SD for metric variables and number and percentage of total in categorical variables*.

## Discussion

The current study is, to our knowledge, the first to compare the performance and 30-day outcome of the latest-generation S3U with the third-generation, well-established S3 in a modern primary TAVR implantation approach in an equal device size distribution between the TAVR generations. This study is also the first to report a comparison using the same delivery system for both TAVR generations, thus excluding major bias to outcome and complications at 30 days.

The main finding and addressed primary endpoint of the study was a significantly lower incidence of paravalvular regurgitation after TAVR with the novel S3U. Furthermore, procedural performance in terms of fluoroscopy time, amount of contrast and rate of post-dilatation were superior compared to the third-generation valve model. Additionally, more patients receiving the S3U were free of symptoms at 30 days follow-up. Overall, the S3U was non-inferior in procedural safety compared to the third-generation prosthesis.

The S3U has been introduced in November 2018 in the available sizes 20, 23, and 26 mm. The novel prosthesis differs by ~40% taller, textured polyethylene terephthalate outer skirt compared to the prior S3. The design changes have been influenced in observation of the very good PVR performance of the 29 mm S3 (21). Hence, the novel S3U prosthesis is only available for patients with an annulus diameter <26.5 mm. Recently, Saia et al. reported their first multicenter prospective registry experience with the novel S3U (11). Furthermore, Rheude et al. (13) and Moriyama et al. (12) presented a comparison of the novel Sapien 3 ultra prothesis with its previous model Sapien 3. Overall, these two least studies and our current work all show a low incidence rate of all major complications both in-hospital and at 30 days proving the safety of the novel device. Though, the patient collective and the procedural characteristics show substantial differences between our current work and the studies by Rheude et al. (13) and Moriyama et al. (12). Our procedural strategy included a modern implantation approach with attempted primary TAVR prosthesis implant leading to a large difference in the rate of predilatation between our study and the other two reports. Only 10.5% of patients were predilatated in this study. In contrast, this rate was as high as ~60% in the study by Rheude et al. (13) and ~34% by Moriyama et al. (12). Furthermore, our study design included only patients suitable for a prosthesis size of 20, 23, and 26 mm (available in both S3 und S3U) as there is no Ultra version available for the 29 mm prosthesis. In the reports by Rheude et al. (13) and Moriyama et al. (12), patients receiving a 29 mm S3 prosthesis accounted for up to a third of the cohort. As already shown in previous studies, the number of patients sized for a 20 mm valve is very low. In our real-world study, there was no S3U 20 mm and only two S3 20 mm prosthesis implantations which we decided to exclude from the study in the interest of comparability.

In our study there were also no differences in the applied delivery system excluding potential implant success bias between the generations evaluated. The other studies used at least in parts the novel Ultra delivery system with the 14 French seamless, expandable Axela® sheath, which was supposed to streamline the procedure, eliminate valve alignment and flex catheter retraction steps. However, in some instances potential issues with both devices were reported. Therefore, the manufacturer has now paired the S3U valve with the conventional Commander Delivery System and 14F eSheath (11). This system is well-established and has been applied routinely with the S3 since 2014. In the current study, we consequently applied the Commander delivery system for both S3 and S3U. This allowed a true evaluation of the prosthesis performance, which was not further confounded by a novel delivery kit. As expected, we found no difference in bleeding rates and access site complications between the two study arms. Finally, we included patients with a mean age of 81.4 ± 8.3 years across all risk categories with a mean STS score of 5.0 ± 4.1 representing an intermediate risk, elderly real-world cohort. In contrast, the patients included by Rheude et al. (13) and Moriyama et al. (12) ranged with a similar mean age demonstrating a low risk group cohort (mean STS between 3 and 4%).

The primary endpoint of our comparison study was the occurrence of PVR after TAVR. Moderate or severe PVR was observed in 1–14% of patients after TAVR, whereas mild PVR ranges from 6 to 63% (5–8, 22). Moderate or severe PVR after TAVR has been consistently shown to increase mid- and long-term mortality (6–8, 21, 23). Several reports have also suggested a worse outcome for even mild PVR (7–9, 22). Numerous factors have been identified to favor the risk for PVR. Among the patient-related factors, aortic annulus and root calcification are the ones most predictive for later PVR (5, 24). Technical-related causes include undersizing of the prosthesis, malpositioning or incomplete apposition to the native annulus (5, 16, 21). Furthermore, balloon-expandable TAVR prostheses are less prone to cause PVR due to specific design and mechanical properties (10). The recent SOLVE trial reported an at least moderate paravalvular regurgitation of 3.4% for the new-generation self-expanding TAVR prosthesis and a 1.5% rate of at least moderate PVR for the third-generation balloon-expandable S3 (25). Pibarot et al. reported for the third-generation S3 a 3.5% rate of at least moderate PVR and a 32.6 and 8.2% rate of mild or mild-moderate PVR, respectively (21). For the latest generation S3U, Saia et al. showed a 10% rate of mild PVR and a 1.4% rate of moderate PVR (11). Rheude et al. (13) reported a significant decrease in the rate of mild PVR with the S3U (19%) compared to the S3 prosthesis (43%). Similarly, Moriyama et al. (12) showed significantly lower rates of mild PVR with the S3U (7%) compared to the S3 prosthesis (19%). In both studies, there was no significant difference in the rate of moderate PVR between the two prothesis. This is in line with our present results showing an 11% rate of mild PVR and a 0.7% rate of moderate PVR for the S3U leading to a significant risk-reduction for PVR compared to the previous S3 model (11.9 vs. 48.5%, *p* < 0.001) which already proved to exert a rather low risk for PVR among other prosthesis types (25, 26). Additionally, the rate of PVR directly after valve deployment was significantly lower in the S3U resulting in a significant lower rate of post-dilatation. Although we did not observe a difference in terms of safety outcome, post-dilatation should be reduced to a minimum to avoid the risk of annulus rupture, rhythm disturbances or valve dislocation. Among the analyzed parameters in the present study, prosthesis-type and prosthesis undersizing were the only two independent predictors for PVR after TAVR. Patients receiving a S3U reported more often relief of symptoms after 30 days which may be attributed to a lower rate of mild PVR. However, long-term outcome with this new S3U will have to be assessed in future studies.

In addition to the above-mentioned reduction in PVR, a superior performance of the new S3U was also observed by a reduced amount of required contrast and fluoroscopy time. We assume that this reduction might partially be a result of the lower rate of post-dilatation. Reduction in contrast to a required minimum is important to prevent peri-procedural renal failure. Large trials have shown rates of 5–57% of patients who suffered from acute kidney injury after TAVR which significantly affects long-term outcome and mortality (27, 28). We observed in-hospital acute kidney failure of stage 2 or higher in 2.0% of patients. However, the rate did not significantly differ between the two cohorts. With the opening of TAVR indication for younger and low-risk patients, approaches to lower radiation exposure become increasingly important. Here, we found a 15% decrease of fluoroscopy time with the latest-generation S3U compared to its third-generation pre-model. Long-term effects will have to be evaluated in further studies.

Compared to other recent publications, we observed a relatively low rate of post-procedural PPI with a tendency to less PPI in the S3U cohort (5.6 vs. 11.0%, *p* = 0.10) at in-hospital assessment (1, 25, 26, 29, 30). Yet, multivariate analysis revealed only preexisting RBBB to be an independent predictor of PPI and thus, a further significant influence of the S3U could not be determined.

Several limitations have to be acknowledged. First, this study reflects “real world” patients across all risk-categories enrolled in a high-volume single-center observational study rather than a highly selected trial population. Although single-center studies represent an institutional performance, they also minimize cofounders. The study was not designed in randomized fashion, but patients were assigned to either the S3 or the S3U cohort depending on their time of treatment. The 143 S3U patients represented the first patients being treated with the new valve. Though there was no experience at the start of enrollment with this new prosthesis, we observed no inferiority in terms of safety but even a superior procedural performance in some aspects compared to the established S3. As we present a real-world study, we did not pre-determine a certain number for each prosthesis size to be implanted. Furthermore, the current study provides only 30-day follow-up data which limits to conclude on hard outcomes.

## Conclusions

This present real-world study shows that the latest-generation balloon-expandable S3U reduces PVR to a minimum in comparison to the previous S3 model, thereby demonstrating the efficacy of improved sealing. A significant decrease of paravalvular regurgitation with this prosthesis provides a promising result given the steadily growing numbers of TAVR procedures. A superiority for the novel valve was also found in terms of post-dilatation rate, radiation time and the amount of contrast as well as relief of symptoms at 30 days. Further studies need to assess whether the reduction of PVR with S3U may have prognostic significance.

## Data Availability Statement

The raw data supporting the conclusions of this article will be made available by the authors, without undue reservation.

## Ethics Statement

The study was approved by the Institutional Review Board (reference number: 2019-14692). The patients/participants provided their written informed consent to participate in this study.

## Author Contributions

AT: conceptualization, project administration, formal analysis and writing—review and editing. MH: conceptualization, formal analysis, and writing—original draft. MG: investigation and formal analysis. FK: investigation and data curation. JR and MS: data curation and methodology. TR, AK, and AB-F: investigation. TM and RvB: supervision and writing—review and editing. All authors have participated in the work and have read and approved the manuscript.

## Conflict of Interest

AT reports having received lecture honoraria from Edwards Lifesciences and Medtronic. FK reports having received consultancy and lecture honoraria from Abbott Cardiovascular and Edwards Lifesciences. TR reports having received lecture honoraria from Abbott Cardiovascular. AB-F reports having received consultancy and lecture honoraria from Abbott Cardiovascular and Edwards Lifesciences. RvB reports having received consultancy and lecture honoraria from Abbott Cardiovascular and Edwards Lifesciences. The remaining authors declare that the research was conducted in the absence of any commercial or financial relationships that could be construed as a potential conflict of interest.

## Abbreviations

AS: aortic stenosis
PPI: permanent pacemaker implantation
PVR: paravalvular regurgitation
RBBB: right bundle branch block
S3: SAPIEN 3 valve prosthesis
S3U: SAPIEN 3 Ultra valve prosthesis
TAVR: transcatheter aortic valve replacement
VARC-2: updated criteria of the Valve Academic Research Consortium.

## References

[1] Mack MJ Leon MB Thourani VH Makkar R Kodali SK Russo M. Transcatheter aortic-valve replacement with a balloon-expandable valve in low-risk Patients. N Engl J Med. (2019) 380:1695–705. 10.1056/NEJMoa1814052 30883058

[2] Popma JJ Deeb GM Yakubov SJ Mumtaz M Gada H O'Hair D. Transcatheter aortic-valve replacement with a self-expanding valve in low-risk patients. N Engl J Med. (2019) 380:1706–15. 10.1056/NEJMoa1816885 30883053

[3] Reardon MJ Van Mieghem NM Popma JJ Kleiman NS Sondergaard L Mumtaz M. Surgical or transcatheter aortic-valve replacement in intermediate-risk patients. N Engl J Med. (2017) 376:1321–31. 10.1056/NEJMoa1700456 28304219

[4] Leon MB Smith CR Mack MJ Makkar RR Svensson LG Kodali SK. Transcatheter or surgical aortic-valve replacement in intermediate-risk patients. N Engl J Med. (2016) 374:1609–20. 27040324 10.1056/NEJMoa1514616

[5] Ong G Annabi MS Clavel MA Guzzetti E Salaun E Toubal O. Paravalvular regurgitation after transcatheter aortic valve replacement: is the problem solved? Interv Cardiol Clin. (2018) 7:445–58. 10.1016/j.iccl.2018.06.005 30274611

[6] Mack MJ Leon MB Smith CR Miller DC Moses JW Tuzcu EM. 5-year outcomes of transcatheter aortic valve replacement or surgical aortic valve replacement for high surgical risk patients with aortic stenosis (PARTNER 1): a randomised controlled trial. Lancet. (2015) 385:2477–84. 10.1016/S0140-6736(15)60308-7 25788234

[7] Makkar RR Thourani VH Mack MJ Kodali SK Kapadia S Webb JG. Five-year outcomes of transcatheter or surgical aortic-valve replacement. N Engl J Med. (2020) 382:799–809. 10.1056/NEJMoa1910555 31995682

[8] Athappan G Patvardhan E Tuzcu EM Svensson LG Lemos PA Fraccaro C. Incidence, predictors, and outcomes of aortic regurgitation after transcatheter aortic valve replacement: meta-analysis and systematic review of literature. J Am Coll Cardiol. (2013) 61:1585–95. 10.1016/j.jacc.2013.01.047 23500308

[9] Laakso T Laine M Moriyama N Dahlbacka S Airaksinen J Virtanen M. Impact of paravalvular regurgitation on the mid-term outcome after transcatheter and surgical aortic valve replacement. Eur J Cardio Thoracic Surg. (2020) 58:1145–52. 10.1093/ejcts/ezaa254 33057657

[10] Abdel-Wahab M Neumann FJ Mehilli J Frerker C Richardt D Landt M. 1-year outcomes after transcatheter aortic valve replacement with balloon-expandable versus self-expandable valves: results from the CHOICE randomized clinical trial. J Am Coll Cardiol. (2015) 66:791–800. 10.1016/j.jacc.2015.06.026 26271061

[11] Saia F Gandolfo C Palmerini T Berti S Doshi SN Laine M. In-hospital and thirty-day outcomes of the SAPIEN 3 Ultra balloon-expandable transcatheter aortic valve: the S3U registry. EuroIntervention. (2020) 15:1240–7. 10.4244/EIJ-D-19-00541 31763985

[12] Moriyama N Lehtola H Miyashita H Piuhola J Niemela M Laine M. Hemodynamic comparison of transcatheter aortic valve replacement with the SAPIEN 3 Ultra versus SAPIEN 3: the HomoSAPIEN registry. Catheterization Cardiovasc Intervent. (2020). 10.1002/ccd.29281. [Epub ahead of print]. 32966682 PMC8247002

[13] Rheude T Pellegrini C Lutz J Alvarez-Covarrubias HA Lahmann AL Mayr NP. Transcatheter aortic valve replacement with balloon-expandable valves: comparison of SAPIEN 3 ultra versus SAPIEN 3. JACC Cardiovasc Intervent. (2020) 13:2631–38. 10.1016/j.jcin.2020.07.013 33129822

[14] Blanke P Weir-McCall JR Achenbach S Delgado V Hausleiter J Jilaihawi H. Computed tomography imaging in the context of transcatheter aortic valve implantation (TAVI)/transcatheter aortic valve replacement (TAVR): an expert consensus document of the society of cardiovascular computed tomography. JACC Cardiovasc Imaging. (2019) 12:1–24. 10.1016/j.jcmg.2018.12.003 30621986

[15] Watanabe Y Morice MC Bouvier E Leong T Hayashida K Lefevre T. Automated 3-dimensional aortic annular assessment by multidetector computed tomography in transcatheter aortic valve implantation. JACC Cardiovasc Intervent. (2013) 6:955–64. 10.1016/j.jcin.2013.05.008 23954060

[16] Willson AB Webb JG Labounty TM Achenbach S Moss R Wheeler M. 3-dimensional aortic annular assessment by multidetector computed tomography predicts moderate or severe paravalvular regurgitation after transcatheter aortic valve replacement: a multicenter retrospective analysis. J Am Coll Cardiol. (2012) 59:1287–94. 10.1016/S0735-1097(12)60326-X 22365423

[17] Willson AB Webb JG Freeman M Wood DA Gurvitch R Thompson CR. Computed tomography-based sizing recommendations for transcatheter aortic valve replacement with balloon-expandable valves: comparison with transesophageal echocardiography and rationale for implementation in a prospective trial. J Cardiovasc Computed Tomogr. (2012) 6:406–14. 10.1016/j.jcct.2012.10.002 23127390

[18] Yang TH Webb JG Blanke P Dvir D Hansson NC Norgaard BL. Incidence and severity of paravalvular aortic regurgitation with multidetector computed tomography nominal area oversizing or undersizing after transcatheter heart valve replacement with the Sapien 3: a comparison with the Sapien XT. JACC Cardiovasc Intervent. (2015) 8:462–71. 10.1016/j.jcin.2014.10.014 25790764

[19] Hahn RT Pibarot P Stewart WJ Weissman NJ Gopalakrishnan D Keane MG. Comparison of transcatheter and surgical aortic valve replacement in severe aortic stenosis: a longitudinal study of echocardiography parameters in cohort A of the PARTNER trial (placement of aortic transcatheter valves). J Am Coll Cardiol. (2013) 61:2514–21. 10.1161/CIRCRESAHA.116.307972 23623915 PMC3931006

[20] Kappetein AP Head SJ Genereux P Piazza N van Mieghem NM Blackstone EH. Updated standardized endpoint definitions for transcatheter aortic valve implantation: the Valve Academic Research Consortium-2 consensus document. J Am Coll Cardiol. (2012) 60:1438–54. 10.1016/j.jacc.2012.09.001 23036636

[21] Pibarot P Hahn RT Weissman NJ Arsenault M Beaudoin J Bernier M. Association of paravalvular regurgitation with 1-year outcomes after transcatheter aortic valve replacement with the SAPIEN 3 valve. JAMA Cardiol. (2017) 2:1208–16. 10.1001/jamacardio.2017.3425 28973091 PMC5710359

[22] Ando T Briasoulis A Telila T Afonso L Grines CL Takagi H. Does mild paravalvular regurgitation post transcatheter aortic valve implantation affect survival? A meta-analysis. Catheterization Cardiovasc Intervent. (2018) 91:135–47. 10.1002/ccd.27336 28963761

[23] Abdel-Wahab M Zahn R Horack M Gerckens U Schuler G Sievert H. Aortic regurgitation after transcatheter aortic valve implantation: incidence and early outcome. Results from the German transcatheter aortic valve interventions registry. Heart. (2011) 97:899–906. 10.1136/hrt.2010.217158 21398694

[24] Khalique OK Hahn RT Gada H Nazif TM Vahl TP George I. Quantity and location of aortic valve complex calcification predicts severity and location of paravalvular regurgitation and frequency of post-dilation after balloon-expandable transcatheter aortic valve replacement. JACC Cardiovasc Intervent. (2014) 7:885–94. 10.1016/j.jcin.2014.03.007 25147034

[25] Thiele H Kurz T Feistritzer HJ Stachel G Hartung P Eitel I. Comparison of newer generation self-expandable vs. balloon-expandable valves in transcatheter aortic valve implantation: the randomized SOLVE-TAVI trial. Eur Heart J. (2020). 10.1093/eurheartj/ehaa036 32049283

[26] Barbanti M Buccheri S Rodes-Cabau J Gulino S Genereux P Pilato G. Transcatheter aortic valve replacement with new-generation devices: a systematic review and meta-analysis. Int J Cardiol. (2017) 245:83–9. 10.1016/j.ijcard.2017.07.083 28760396

[27] Ram P Mezue K Pressman G Rangaswami J. Acute kidney injury post-transcatheter aortic valve replacement. Clin Cardiol. (2017) 40:1357–62. 10.1002/clc.22820 29251358 PMC6490570

[28] Thongprayoon C Cheungpasitporn W Gillaspie EA Greason KL Kashani KB. The risk of acute kidney injury following transapical versus transfemoral transcatheter aortic valve replacement: a systematic review and meta-analysis. Clin Kidney J. (2016) 9:560–6. 10.1093/ckj/sfw055 27478597 PMC4957730

[29] Vlastra W Chandrasekhar J Munoz-Garcia AJ Tchetche D de Brito FS Jr Barbanti M. Comparison of balloon-expandable vs. self-expandable valves in patients undergoing transfemoral transcatheter aortic valve implantation: from the CENTER-collaboration. Eur Heart J. (2019) 40:456–65. 10.1093/eurheartj/ehy805 30590565

[30] Ben-Shoshan J Konigstein M Zahler D Margolis G Chorin E Steinvil A. Comparison of the edwards SAPIEN S3 versus medtronic evolut-R devices for transcatheter aortic valve implantation. Am J Cardiol. (2017) 119:302–7. 10.1016/j.amjcard.2016.09.030 28029363

