# Supplementary material for: Minimizing Paravalvular Regurgitation With the Novel SAPIEN 3 Ultra TAVR Prosthesis: A Real-World Comparison Study
Source: Front Cardiovasc Med. 2021 Mar 18;8:623146. doi: 10.3389/fcvm.2021.623146 (PMC8015438; doi:10.3389/fcvm.2021.623146)
Supplement: Supplementary file 1 [file Data_Sheet_1.docx]

**Table S1 Antithrombotic therapy at baseline**

|  | **All Patients (N = 343)** | **Sapien 3 (N = 200)** | **Sapien 3 Ultra (N = 143)** | **p value** |
| --- | --- | --- | --- | --- |
| No antithrombotic therapy n (%) | 101 (29.4) | 55 (27.5) | 46 (32.2) | 0.350 |
| Aspirine monotherapy n (%) | 36 (10.5) | 24 (12.0) | 12 (8.4) | 0.282 |
| Aspirine + P2Y12 inhibitor n (%) | 104 (30.3) | 61 (30.5) | 43 (30.1) | 0.932 |
| DOAC monotherapy n (%) | 47 (13.7) | 25 (12.5) | 22 (15.4) | 0.444 |
| DOAC + P2Y12 inhibitor n (%) | 36 (10.5) | 21 (10.5) | 15 (10.5) | 0.998 |
| Coumarin monotherapy n (%) | 8 (2.3) | 6 (3.0) | 2 (1.4) | 0.333 |
| Coumarin + P2Y12 inhibitor n (%) | 11 (3.2) | 8 (4.0) | 3 (2.1) | 0.324 |

DOAC = direct oral anticoagulants

P value was calculated by Chi squared test. Data represented as percentage of total.

**Table S2 MSCT annular measurements and oversizing**

|  | **All Patients (N = 343)** | **Sapien 3 (N = 200)** | **Sapien 3 Ultra (N = 143)** | **p value** |
| --- | --- | --- | --- | --- |
| Annulus area (mm²) | 443.4 ± 58.7 | 442.2 ± 58.6 | 445.2 ± 58.9 | 0.632 |
| 23mm SAPIEN | 397.7 ± 33.6 | 398.8 ± 32.6 | 395.9 ± 25.2 | 0.575 |
| 26mm SAPIEN | 491.6 ± 36.7 | 491.0 ± 40.1 | 492.5 ± 32.0 | 0.789 |
| Annulus diameter mean (mm) | 23.7 ± 1.6 | 23.7 ± 1.6 | 23.8 ± 1.6 | 0.637 |
| 23mm SAPIEN | 22.5 ± 1.0 | 22.6 ± 0.9 | 22.5 ± 1.0 | 0.561 |
| 26mm SAPIEN | 25.0 ± 0.9 | 25.0 ± 1.0 | 25.1 ± 0.8 | 0.749 |
| Oversizing <0% - n (%) | 115 (33.5) | 72 (36.0) | 43 (30.1) | 0.251 |
| Oversizing 0% to 5% - n (%) | 77 (22.4) | 39 (19.5) | 38 (26.6) | 0.122 |
| Oversizing 5% to 10% - n (%) | 65 (19.0) | 41 (20.5) | 24 (16.8) | 0.386 |
| Oversizing >10% - n (%) | 86 (25.1) | 48 (24.0) | 38 (26.6) | 0.588 |

P value was calculated by Chi squared test and Student's t-test. Data represented as mean ± SD for metric variables and number and percentage of total in categorical variables.

**Table S3 Predictors of paravalvular regurgitation**

|  | **Univariate Analysis** |  | **Multivariate Analysis** |  |
| --- | --- | --- | --- | --- |
|  | **Odds Ratio (CI)** | **p value** | **Odds Ratio (CI)** | **p value** |
| Prosthesis (S3U vs S3) | **0.14 (0.80 - 0.26)** | **< 0.001** | **0.13 (0.07 - 0.24)** | **< 0.001** |
| Oversizing* | **0.75 (0.64 - 0.89)** | **0.001** | **0.75 (0.62 - 0.90)** | **0.002** |
| Bicuspid Valve | 0.63 (0.31 - 1.27) | 0.194 | 0.59 (0.27 - 1.30) | 0.172 |
| Severe Commissural Calcification | 1.32 (0.84 - 2.08) | 0.236 | 1.27 (0.75 - 2.16) | 0.330 |
| Age | 1.01 (0.98 - 1.05) | 0.393 | 1.02 (0.98 - 1.06) | 0.371 |
| Sex (male) | 1.29 (0.82 - 2.03) | 0.266 | 1.36 (0.80 - 2.31) | 0.261 |

S3 = SAPIEN 3, S3U = SAPIEN 3 Ultra

*oversizing categories see table S2

P value was calculated by Chi squared test. Data represented as odds ratio and confidence interval.

**Table S4 Predictors of permanent pacemaker implantation**

|  | **Univariate** |  | **Multivariate** |  |
| --- | --- | --- | --- | --- |
|  | **Odds Ratio (CI)** | **p value** | **Odds Ratio (CI)** | **p value** |
| Prosthesis (S3U vs S3) | 0.46 (0.19 - 1.13) | 0.090 | 0.47 (0.18 - 1.22) | 0.120 |
| Oversizing* | 0.85 (0.64 - 1.13) | 0.258 | 0.86 (0.63 - 1.17) | 0.343 |
| Post Dilation | 0.51 (0.07 - 3.97) | 0.523 | 0.52 (0.06 - 4.59) | 0.564 |
| Bicuspid valve | 1.86 (0.71 - 4.89) | 0.205 | 2.16 (0.68 - 6.84) | 0.191 |
| Severe commissural calcification | 2.10 (0.94 - 4.75) | 0.071 | 1.61 (0.65 - 3.97) | 0.141 |
| RBBB preexisting | **9.81 (3.63 - 26.55)** | **< 0.001** | **9.22 (2.75 - 30.96)** | **< 0.001** |
| LBBB pre-existing | 1.77 (0.71 - 4.41) | 0.224 | 1.92 (0.70- 5.30) | 0.209 |
| AVB pre-existing | 2.37 (0.98 - 5.72) | 0.056 | 1.88 (0.67 - 5.31) | 0.232 |
| Age | 1.00 (0.95 - 1.06) | 0.994 | 1.00 (0.94 - 1.06) | 0.762 |
| Sex (male) | 0.80 (0.37 - 1.77) | 0.585 | 0.76 (0.31 - 1.86) | 0.640 |

AVB = Atrioventricular Block °I, LBBB = Left Bundle Branch Block, RBBB = Right Bundle Branch Block, S3 = SAPIEN 3, S3U = SAPIEN 3 Ultra

*oversizing categories table S2

P value was calculated by Chi squared test. Data represented as odds ratio and confidence interval.
